# Schizotypy in Parkinson’s disease predicts dopamine-associated psychosis

**DOI:** 10.1038/s41598-020-80765-5

**Published:** 2021-01-12

**Authors:** Carina R. Oehrn, Jana Schönenkorb, Lars Timmermann, Igor Nenadić, Immo Weber, Phillip Grant

**Affiliations:** 1 Department of Neurology, Philipps-University Marburg, Marburg, Germany; 2 Center for Mind, Brain and Behavior (CMBB), Philipps-University Marburg, Marburg, Germany; 3 Department of Psychiatry and Psychotherapy, Philipps-University Marburg, Marburg, Germany; 4 Psychology School, Fresenius University of Applied Sciences, Frankfurt, Germany; 5 Faculty of Life Science Engineering, Technische Hochschule Mittelhessen University of Applied Sciences, Giessen, Germany; 6 Department of Neurology, University Hospital Giessen and Marburg, Baldingerstrasse, 35043 Marburg, Germany

**Keywords:** Human behaviour, Neurology, Signs and symptoms

## Abstract

Psychosis is the most common neuropsychiatric side-effect of dopaminergic therapy in Parkinson’s disease (PD). It is still unknown which factors determine individual proneness to psychotic symptoms. Schizotypy is a multifaceted personality trait related to psychosis-proneness and dopaminergic neurotransmission in healthy subjects. We investigated whether (1) PD patients exhibit lower schizotypy than controls and (2) dopamine-related neuropsychiatric side-effects can be predicted by higher schizotypy. In this cross-sectional study, we used the Oxford-Liverpool Inventory of Feelings and Experiences in 56 PD patients (12 women, mean ± sd age: 61 ± 11 years) receiving their usual dopaminergic medication and 32 age-matched healthy controls (n = 32; 18 women, mean ± sd age: 57 ± 6 years). We further compared schizotypy scores of patients with (n = 18, 32.1%) and without previously experienced psychosis. We found that patients exhibited lower schizotypy than controls. Further, patients with a history of psychosis exhibited higher schizotypy than patients without these symptoms. Using an information theoretic measure and a machine learning approach, we show that schizotypy yields the greatest predictive value for dopamine-associated hallucinations compared to other patient characteristics and disease related factors. Our results indicate an overlap between neural networks associated with schizotypy and the pathophysiology of PD and a relationship between schizotypy and psychotic side-effects of dopaminergic medication.

## Introduction

Parkinson’s disease (PD) is the second most common neurodegenerative disease worldwide^1^. In high-income countries, PD affects 160 per 100,000 people aged 65 years or older^2^. PD is usually treated with dopaminergic medication, most commonly with dopamine agonists and levodopa. Dopaminergic treatment is associated with a range of possible motor and non-motor side-effects, which can limit the therapeutic window and cause a considerable burden for patients and their care givers^3,4^. Psychosis is the most common neuropsychiatric side-effect of dopaminergic therapy in PD and occurs in approximately one-third of patients^5,6^. PD psychosis is characterized by recurrent hallucinations, illusions, false sense of presence or delusions after disease onset that are not attributable to any other psychiatric or general medical conditions^7,8^. Hallucinations, i.e. spontaneous aberrant perceptions, and illusions, i.e. misinterpretations of perceived real stimuli, most frequently occur in visual (~ 22–38%) and auditory domains (~ 8–22%) during the course of dopaminergic treatment^5,9^. Delusions are less common in PD patients (1–7%) and primarily involve jealousy, persecution and abandonment^5,6,10,11^. Psychosis in untreated PD patients is rare (1–3%)^12–14^.

It is still unknown which factors determine individual proneness to neuropsychiatric side effects of dopamine. Several studies have demonstrated that dopamine-associated hallucinations cannot be explained by mere medication-dose or drug-class which suggests a multi-factorial pathogenesis involving direct medication effects, disease-related elements and other risk factors^6,11,15^. While studies consistently establish a relationship between cognitive impairment and the occurrence of psychosis in advanced PD, cognitive decline does not account for the occurrence of hallucinations in early PD^10^. Further, evidence for the independent contribution of age, disease duration, disease severity and comorbidities, such as depression, for the development of hallucinations remain inconclusive^6,10,11,15–17^. Thus, it is still unclear which phenotype predisposes towards PD psychosis.

Human neuroimaging studies suggest that behavioral effects of administered dopamine depend on individual baseline dopamine levels and fronto-striato-thalamic connectivity e.g.^18^. Recent studies in healthy subjects have demonstrated that enhanced dopaminergic activity in the brain is associated with higher positive schizotypy^19^. Schizotypy describes a constellation of personality traits with four dimensions measuring perceptual aberrations and unusual ideation (positive dimension), reductions in emotional, motivational, physical and social function (negative dimension), cognitive or associated slippage related to formal thought disorder (disorganized dimension), as well as eccentric, impulsive and nonconformist behavior and speech patterns (impulsive/nonconformist dimension)^20,21^. Of these facets, positive schizotypy has been linked to proneness for psychosis as well as for psychotic-like experiences in healthy individuals, while negative and disorganized schizotypy are related to schizophrenia-liability e.g.^22–24^.

Motor as well as non-motor symptoms of PD are associated with a loss of dopaminergic neurons in the midbrain and a disruption of basal ganglia-thalamo-cortical processing^25^. Similarly, variations in schizotypy are mediated by dopamine and associated with structural and functional alterations in basal ganglia-thalamo-cortical networks^26–29^. The relationship between schizotypy and dopamine release is, however, inverse compared to PD. The expression of schizotypy in PD patients compared to healthy controls is still unknown. Previous studies comparing small cohorts of PD patients (n = 18–26) to controls observed either decreased^30^ or increased positive schizotypy in PD patients^31^ or no difference between groups^32^. Further, it is still unclear, whether variations in schizotypy explain PD patients’ proneness to neuropsychiatric dopaminergic side-effects. Based on the above, we hypothesize that PD patients exhibit lower positive schizotypy. Within patients, however, we assume that those prone to psychotic side effects of dopaminergic medication have relatively higher values in positive schizotypy.

In this study, we compare schizotypy scores of 56 PD patients to age-matched healthy controls. Furthermore, we investigate whether PD patients with higher values in positive schizotypy are more prone to the psychotic side effects of dopaminergic medication. The identification of risk factors for dopamine-associated non-motor complications is an essential step towards optimization and individualization of PD patient treatment by potentially decreasing the debilitating side effects associated with dopaminergic medication in a significant portion of PD patients.

## Methods

### Patients

In this cross-sectional study, we included a total of 56 PD patients at all stages of disease severity (Table 1). We chose this study design in order to avoid selection bias by matching parameters, e.g. as could occur in a case–control study, and possible over-matching and therefore enable a fully data-driven, multifactorial prediction of psychosis. We excluded patients with other relevant neurological and psychiatric diseases including dementia and depression. In order to maximize external validity of our results, we did not otherwise restrict the patient population to particular subject characteristics or disease-related factors. We did not screen patients or controls for family history of psychiatric disorders.

**Table 1:** Demographics and clinical data.

	Controls	PD patients		
		Overall	Psychosis	No psychosis
Number of subjects	32	56	18 (32.1%)	38 (67.9%)
**Demographics**				
Age (years)	57.4 ± 5.5	61.0 (± 11.0)	63.2 ± 12.8	59.9 ± 10.1
Sex (female)	18 (56.3%)	12 (21.4%)	2 (11.1%)	10 (26.3%)
**Clinical data**				
Disease duration (years)	N/A	8.5 ± 5.1	7.9 ± 4.4	8.7 ± 5.4
Hoehn & Yahr stage (median)	N/A	2	2	2
Subtype: akinetic-rigid	N/A	24 (42.9%)	8 (44.4%)	16 (42.1%)
Subtype: equivalent	N/A	19 (33.9%)	7 (38.9%)	12 (31.6%)
Subtype: tremor-dominant	N/A	13 (23.2%)	3 (16.7%)	10 (26.3%)
LEDD (mg)	N/A	917.6 ± 1051.1	1151.8 ± 1658.8	806.7 ± 582.2
Total UPDRS	N/A	52.5 ± 18.2	60.0 ± 19.0	48.9 ± 16.9
UPDRS I	N/A	11.9 ± 6.2	15.7 ± 6.3	10.1 ± 5.4
UPDRS II	N/A	13.7 ± 7	15.9 ± 8.9	12.6 ± 5.8
UPDRS III	N/A	26.9 ± 11.9	28.3 ± 11.2	26.2 ± 12.3
**Schizotypy scores**				
UnEx	7.4 ± 6.8	4.1 ± 4.0	7.1 ± 4.5	2.8 ± 2.9
UnEx_corr_	N/A	3.5 ± 3.5	5.6 ± 4.1	2.5 ± 2.6
CogDis	8.3 ± 6.4	8.1 ± 5.6	9.2 ± 6.0	7.6 ± 5.4
IntAn	6.9 ± 4.9	8.1 ± 5.0	7.1 ± 4.6	8.7 ± 5.2
ImpNon	6.8 ± 3.3	4.6 ± 2.1	5.3 ± 2.6	4.2 ± 1.8

If not otherwise indicated mean ± SD or n (%) of patients.

Patients were recruited from the outpatient clinic and the ward of the Department of Neurology of the University Hospital Marburg within a period of one year and diagnosed according to the Movement Disorder Society diagnostic criteria^33^. We obtained all data while patients received their medication as usual (medication ON).

Clinical severity was assessed using the Unified Parkinson’s Disease Rating Scale (UPDRS, version 2008) including non-motor experiences of daily living (UPDRS I), motor experiences of daily living (UPDRS II) and motor examination (UPDRS III). The UPDRS III was obtained by two independent clinicians. The study protocol was approved by the medical ethics committee Marburg and conducted in accordance with the latest version of the Declaration of Helsinki. All patients were mentally and physically capable to provide written informed consent. The written informed consent was signed from all participants included in this study.

### Assessment of hallucinations and psychosis

We assessed psychosis retrospectively in a structured clinical interview. First, a clinician evaluated current symptoms by directly asking patients according to item 1.2 of the UPDRS I (v.2008), a common tool for the assessment of PD psychosis (“Over the past week, have you seen, heard, smelled or felt things that were not really there?”)^8^. In this question, the clinician probes the occurrence of illusions, hallucinations, as well as delusions. Symptom severity is rated based on the quality of symptoms, patients’ insight and the presence of delusions and paranoia (0 = normal: no hallucinations or psychotic behavior; 1 = slight: illusions or non-formed hallucinations, but patient recognizes them without loss of insight; 2 = formed hallucinations independent of environmental stimuli; 3 = moderate: formed hallucinations with loss of insight; 4 = severe: patient has delusions or paranoia). Loss of insight and delusions were evaluated by means of addressing family members or care-takers. Whenever a second person was not available, the clinician evaluated the symptoms within the social and personal context of the patient established throughout the clinical interview. In regard to delusions, patients were asked whether they had the feeling of persecution and if they had the impression that people refer to or intend to harm them. We considered formed hallucinations for the subsequent analysis (UPDRS I, item 1.2. > 1). Second, the clinician assessed whether patients experienced these symptoms at any time point during the course of the disease. Finally, we asked whether patients had noticed any association between the occurrence of such perceptions and specific medications.

### Assessment of schizotypy

Schizotypy scores were assessed using the German^34^ version of the multidimensional Oxford-Liverpool Inventory of Feelings and Experiences (O-LIFE)^35,36^. The examiner assured that there was no missing data. The O-LIFE comprises four facets of schizotypy including Unusual Experiences (UnEx, positive schizotypy), Introvertive Anhedonia (IntAn, negative schizotypy), Cognitive Disorganization (CogDis, disorganized schizotypy) and Impulsive Nonconformity (ImpNon, impulsive, nonconformist or eccentric features). We used previously published age-matched control data of healthy controls (Table 1)^34^.

### Statistical analyses

We performed all analyses using Matlab (Mathworks, Version 2016b). Due to the relatively small sample size and the violation of normal distribution, we chose non-parametric tests for our analysis. We compared the results of the O-LIFE questionnaire of PD patients to healthy controls using a Wilcoxon signed rank test. To this end, we performed a two-sided test of the hypothesis that the data originates from a distribution with a median equal to the previously reported mean scores of healthy controls^34^. Non-parametric effect sizes were calculated as Cliff’s Delta by means of the measures-of-effect-size-toolbox for Matlab^37^. To this end, we calculated the area under curve of the receiver-operating characteristic (AUROC) and thereafter estimated effect sizes as Cliff’s Delta = 2*AUROC-1.

#### Comparison of schizotypy

Subsequently, we investigated the relationship between schizotypy and dopamine-associated hallucinations. To this end, we split patients into two groups: patients with vs. without respective symptoms. In order to avoid circular argumentation for this analysis, we excluded those items of the O-LIFE that specifically pertained to experiences that were used to classify patients into the aforementioned sub-groups (UnEx_corr_; items OL044, OL071, OL083, OL089, OL090). We divided the data based on results of UPDRS I sub-item 1.2 for current symptoms and based on the clinical interview for symptoms during any time throughout the disease. Thereafter, we investigated differences in schizotypy between groups by means of a Wilcoxon rank sum test for equal medians. Furthermore, we investigated relationships between UnEx/ImpNon scores, age, disease duration, total UPDRS scores (UPDRS I–III) and the levodopa equivalent daily dose of medication (LEDD) by means of Pearson correlations. Albeit that we expected differential findings with regards to schizotypy (i.e. associations should be specific to the positive schizotypy facet), we corrected for multiple comparisons using the false discovery rate procedure adapted by Benjamin and Hochberg^38^. For all analyses, we set an α-level of 0.05.

#### Prediction of current hallucinations

In order to estimate the contribution of different patient characteristics to the multifactorial prediction of hallucinations, we used two methods. First, we calculated predictor importance by means of mutual information (MI)^39^, an information theoretic measure which quantifies the general statistical dependency of two variables, i.e. how well may one variable be predicted when knowing the other^40^. Predictor variables comprised UnEx_corr_, CogDis, IntAn and ImpNon scores, as well as basic patient information, such as age, sex, disease duration, Hoehn and Yahr stage (H&Y) and subtype of PD (akinetic-rigid, tremor-dominant, equivalent subtypes). Additionally, we included LEDD, total UPDRS I (without item 1.2), UPDRS II and UPDRS III scores. As all patient characteristics represent current data, we predicted the occurrence of current hallucinations (as assessed by UPDRS I, item 1.2). MI was estimated using a binning estimator, and bin sizes optimized by applying the Freedman-Diaconis rule^41^. The predictor variables were subsequently sorted with respect to their predictor importance quantified by MI. Second, we used a machine learning approach and classified the incidence of hallucinations based on the same predictors used in the MI analysis. As we aimed for an easily interpretable classifier suited for a mix of categorical and numeric predictors, we tested multiple algorithms including logistic regression, linear support vector machine, decision trees and naïve Bayes. We improved class balance by synthetically creating new examples from the minority class via linear interpolation between existing minority class samples (Synthetic Minority Over-sampling Technique, SMOTE)^42^ creating more examples in the vicinity of the boundary between the two classes than within the interior of the minority class (Adaptive Synthetic Sampling Approach, ADASYN)^43^. We performed fivefold cross-validation; i.e. 20% of the data were used for testing. Thereafter, we used the classifier providing the best fit for our data and performed a jackknife procedure in order to estimate the contribution of each predictor to the accuracy of the model. To this end, we systematically removed each feature from the dataset and recalculated the accuracy of the model. Due to the probabilistic nature of model estimation, we performed this procedure 1000 times per observation and averaged the resulting accuracy values.

## Results

### PD patients exhibit lower schizotypy

#### Comparison of schizotypy between patients and controls

**Figure 1:**
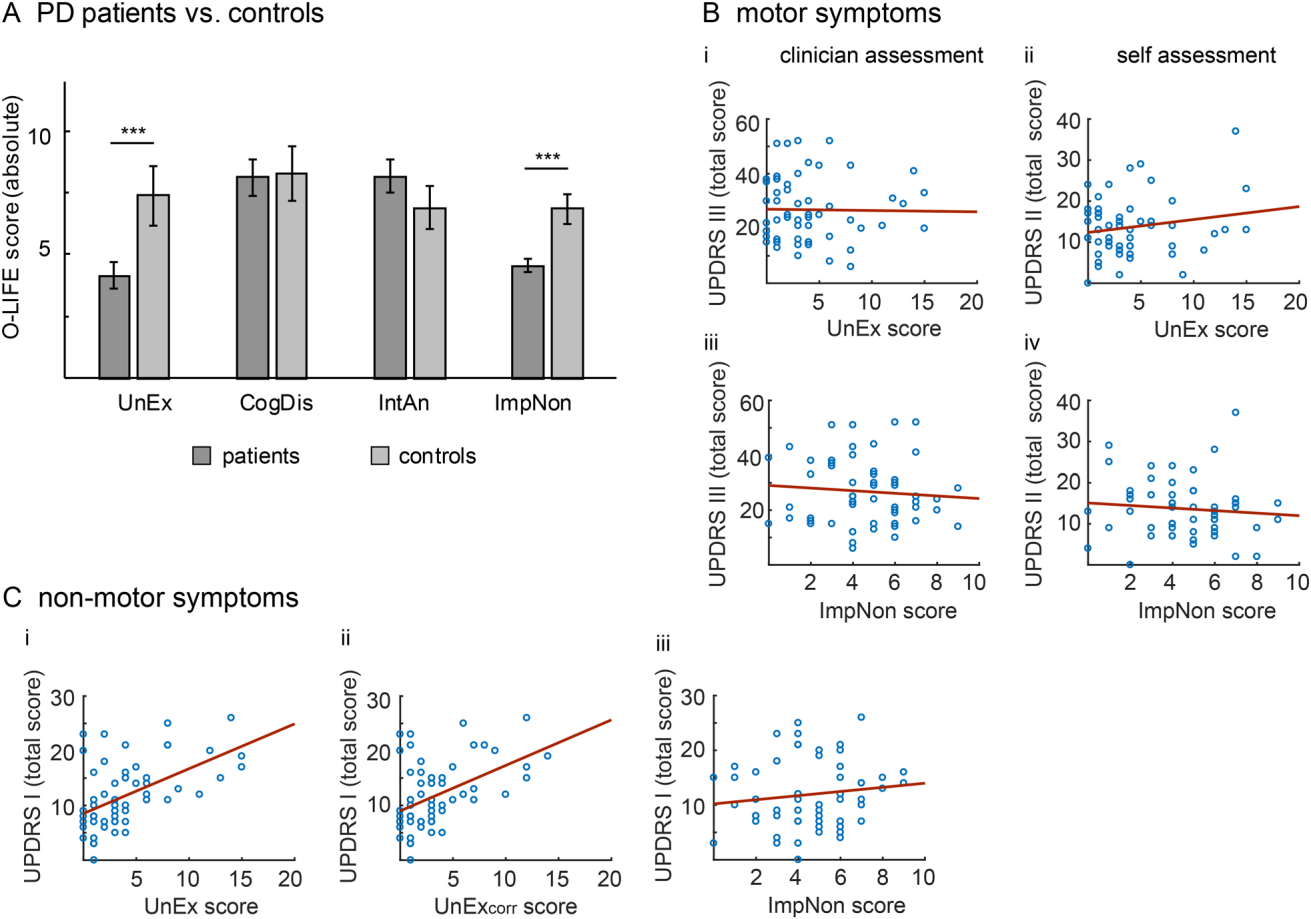
Schizotypy in PD patients compared to controls. (**A**) Mean (± standard error) absolute values of O-LIFE subscores across PD patients and healthy controls. PD patients exhibit lower positive schizotypy than age-matched healthy controls. Results of two-sided Wilcoxon signed rank tests are indicated: ***p < 0.001. (**B**) UnEx and ImpNon scores did not correlate with motor impairment rated by clinicians (UPDRS III; Bi and Biii) or by patients themselves (UPDRS II; Bii and Biv). Blue dots illustrate data from individual patients. (**C**) Illustration of non-motor impairment in PD patients as a function of original (UnEx) and corrected UnEx scores (UnEx_corr_). UPDRS I scores correlated with UnEx (Ci) and UnEx_corr_ (Cii), but not ImpNon scores (Ciii).

Data of all 56 patients were eligible for subsequent analysis. The two-sided Wilcoxon signed rank test showed that median schizotypy of patients was lower than the previously reported mean of age-matched healthy controls (Fig. 1A, Table 1). As hypothesized, this effect was specific to sub-scores of the O-LIFE; i.e., UnEx (p_corr_ < 0.001, Cliff’s Delta = − 0.61) and ImpNon (p_corr_ < 0.001, Cliff’s Delta = − 0.64), while there were no differences for CogDis (p_corr_ = 0.46) and IntAn (p_corr_ = 0.27).

#### Relationship between schizotypy, demographics and clinical data

Wilcoxon signed rank tests did not reveal any differences in UnEx and ImpNon scores between women and men (UnEx: p_corr_ = 0.67; ImpNon: p_corr_ = 0.87), patients with the three different sub types of PD (UnEx: p_corr_ = 0.72; ImpNon: p_corr_ = 0.72) or disease severity as measured by the H&Y scale (UnEx: p_corr_ = 0.37; ImpNon: p_corr_ = 0.72).

Further, UnEx and ImpNon scores did not correlate with age (UnEx: R =  − 0.04, p_corr_ = 0.88; ImpNon: R =  − 0.17, p_corr_ = 0.5), disease duration (UnEx: R = 0.03, p_corr_ = 0.89; ImpNon: R = 0.01, p_corr_ = 0.93), LEDD (UnEx: R = 0.08, p_corr_ = 0.75; ImpNon: R = 0.07, p_corr_ = 0.77), and motor impairment (Fig. 1Bi–iv; UnEx–UPDRS II: R = 0.18, p_corr_ = 0.49; UnEx–UPDRS III: R =  − 0.02, p_corr_ = 0.93; ImpNon–UPDRS II: R =  − 0.09, p_corr_ = 0.75; ImpNon–UPDRS III: R =  − 0.09, p_corr_ = 0.75). However, we found a positive correlation between total UPDRS I and UnEx (Fig. 1Ci; R = 0.53, p_corr_ < 0.001) but not ImpNon scores (Fig. 1Ciii; R = 0.13, p_corr_ = 0.6). The correlations between UnEx and total UPDRS I scores remained significant using the UnEx_corr_ score (Fig. 1Cii, R = 0.46, p_corr_ < 0.01). Correlations between UnEx scores and UPDRS I sub-items revealed a relationship between UnEx scores and specific sub-scores of the UPDRS I. UnEx scores correlated with hallucinations and psychosis scores (item 1.2, R = 0.54, p_corr_ < 0.001), which remained significant after removal of items related to previously experienced misperceptions (UnEx_corr,_ R = 0.45, p_corr_ = 0.01). Further, UnEx scores did not correlate with cognitive impairment (item 1, R = 0.1, p_corr_ = 0.67), but increased with depressed mood (item 3, R = 0.39, p_corr_ = 0.02), daytime sleepiness (item 8, R = 0.42, p_corr_ = 0.01) and light headedness (item 12, R = 0.36, p_corr_ = 0.04). Further, there was a trend towards a significant correlation between UnEx scores and apathy (item 5, R = 0.35, p_corr_ = 0.05), as well as UnEx scores and fatigue (item 13, R = 0.33, p_corr_ = 0.07).

### Hallucinations are associated with higher UnEx scores

#### Comparison of schizotypy between patient groups

**Figure 2:**
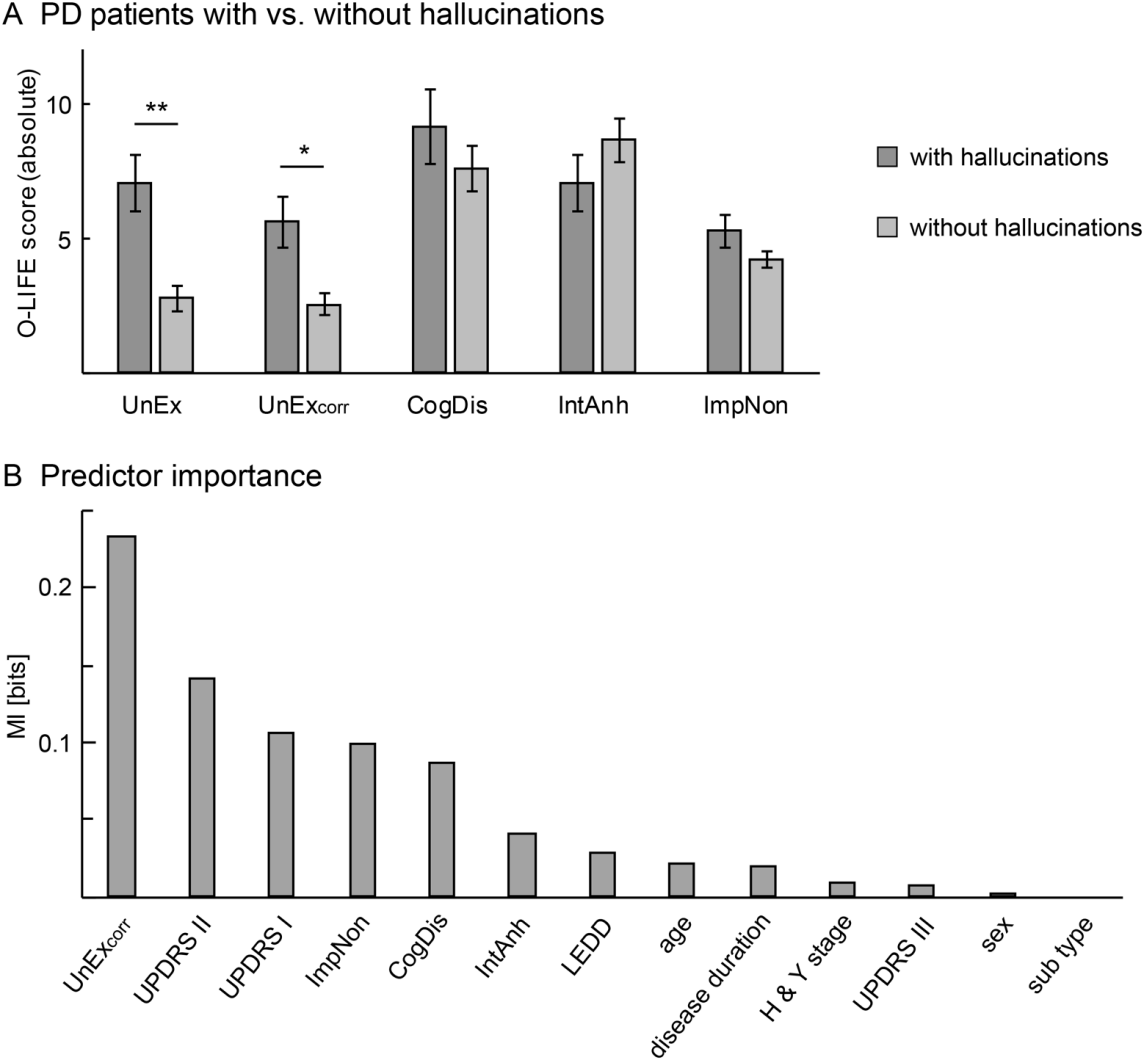
Schizotypy and dopamine-associated hallucinations in PD. (**A**) Mean (± standard error) absolute values of O-LIFE subscores in PD patients with vs. without hallucinations. Patients, who previously experienced hallucinations exhibited higher original (UnEx) and corrected UnEx scores (UnEx_corr_) than patients who had never developed hallucinations. There were no differences in other dimensions of schizotypy. Results of two-sided Wilcoxon rank sum tests are indicated: *p < 0.05, **p < 0.01. (**B**) Estimation of the contribution of different patient characteristics to the prediction of current hallucinations by means of mutual information (MI). UnEx_corr_ scores demonstrated the greatest statistical dependency with the occurrence of hallucinations (MI = 0.23 bits), followed by the UPDRS II (MI = 0.14 bits) and UPDRS I scores (MI = 0.11 bits).

Eighteen patients reported hallucinations throughout the course of the disease (32% of all patients). Twelve of these patients stated that symptoms ceased after a change in the medication regime, and eight patients were able to name the drug eliciting symptoms. Two-sided Wilcoxon rank sum tests revealed that patients who had previously experienced hallucinations exhibited higher UnEx scores than patients, who had never developed those symptoms (Fig. 2A, Table 1; p_corr_ < 0.01, Cliff’s Delta = 0.60). These groups did not differ in CogDis (p_corr_ = 0.31), IntAn (p = 0.29) or ImpNon scores (p_corr_ = 0.11). This effect remained significant using the UnEx_corr_ scores (p_corr_ = 0.028, Cliff’s Delta = 0.46). The groups did not differ in other characteristics, such as age (p_corr_ = 0.85), sex (p_corr_ = 0.85), disease duration (p_corr_ = 1), H&Y stage (p_corr_ = 1), disease subtype (p_corr_ = 1), LEDD (p_corr_ = 1), current severity of motor (UPDRS II: p_corr_ = 0.85; UPDRS III: p_corr_ = 1) and non-motor symptoms (UPDRS I without item 1.2: p_corr_ = 0.096) and incidence of delusions (all p_corr_ > 0.09). Six patients acutely suffered from these symptoms (UPDRS I, item 1.2, score = 2: n = 3; score = 3: n = 3). UnEx_corr_ scores of these six patients did not differ from patients who previously experienced but did not currently present with hallucinations (n = 12; p_corr_ = 0.29). Further, these two groups did not differ in any other patient characteristics (all p_corr_ > 0.84). Six patients (10.7%) reported previous delusions. None of the patients currently suffered from these symptoms. There was no difference in schizotypy between the group with and without previous delusions (UnEx: p_corr_ = 0.68, CogDis: p_corr_ = 0.68, IntAn: p_corr_ = 0.79 and ImpNon: p_corr_ = 0.79).

None of the other sub-items of the UPDRS I correlated with psychosis, as measured by item 2. Importantly, psychosis was not dependent on cognitive impairment (item 1–items 2: R = 0.22, p_corr_ = 0.32). Also, there was no relationship between psychosis and the other UPDRS I items that correlate with schizotypy (depressed mood: item 3–item 2, R = 0.13, p_corr_ = 0.6; daytime sleepiness: item 8–item 2, R = 0.19, p_corr_ = 0.43; light headedness: item 12–item 2, R = 0.3, p_corr_ = 0.12).

#### Prediction of current hallucinations

**Figure 3:**
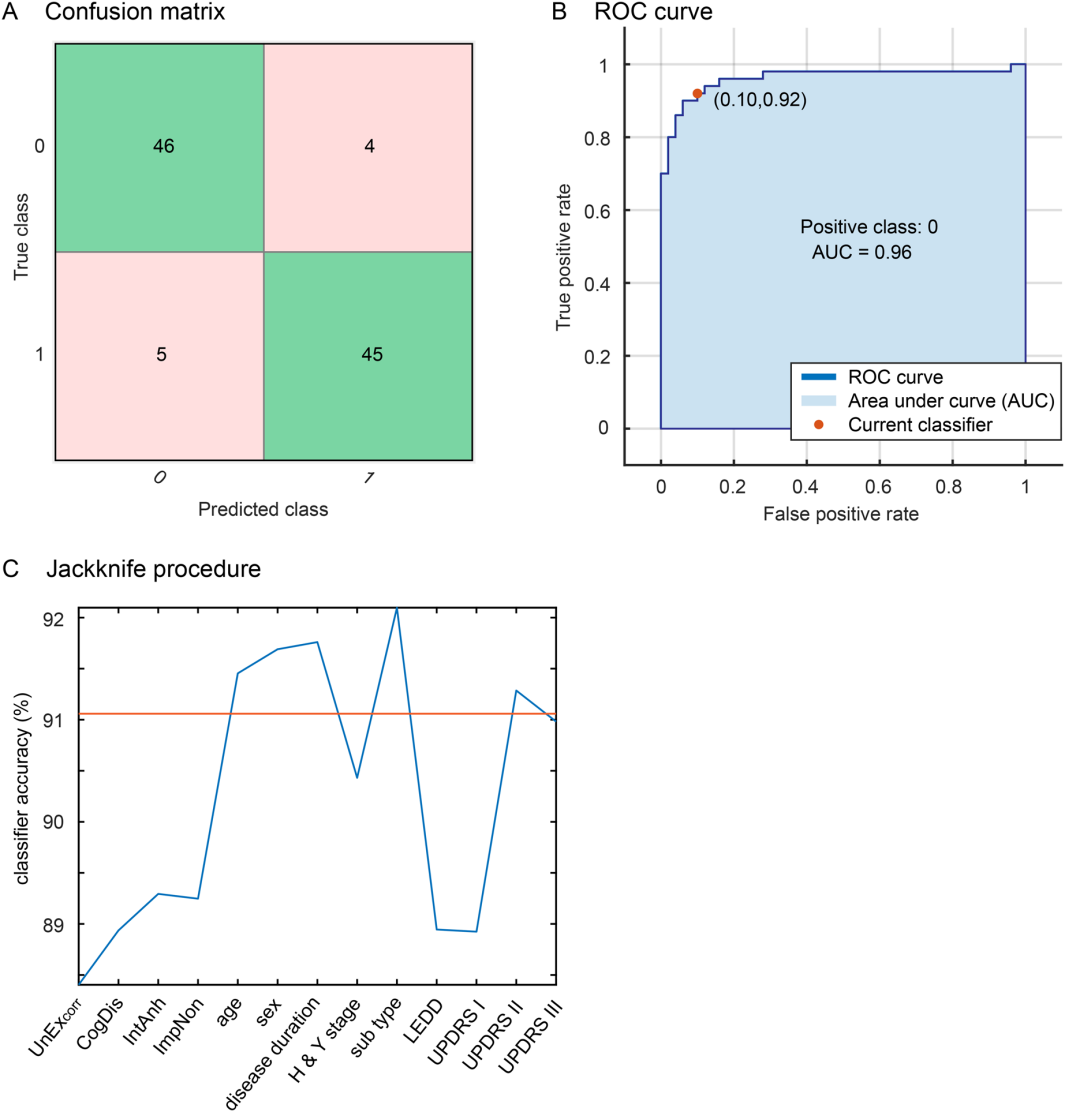
Prediction of hallucinations using machine learning. (**A**) Confusion matrix of Naïve Bayes classifier using all predictors. One indicates current hallucinations and zero no current hallucinations. (**B**) Receiver-operator curve (ROC) illustrating the true positive rate as a function of false positive rate for the whole parameter space. The red dot indicates the optimal classifier shown in (**A**). (**C**) Jackknife procedure showing averaged classifier accuracy across 1000 classifications as a function of removed predictors. The largest change in classifier performance is observed after removing corrected UnEx scores (88.4%). The red line indicates average original classifier accuracy across 1000 classifications including all items (91.1%).

The analysis of MI and the machine learning approach provided converging evidence that positive schizotypy bears the highest predictor importance for the development of hallucinations among patient characteristics and disease-related factors. The UnEx_corr_ score ranked top for the MI (Fig. 2B, MI = 0.23 bits). The next two important variables were total UPDRS II (MI = 0.14 bits) and UPDRS I (MI = 0.11 bits) scores. This was confirmed by our machine learning approach. Gaussian naïve Bayes classification provided the best fit for our data including the 13 predictors (Fig. 3A,B; mean accuracy across 1000 classifications = 91.1%; area under the curve = 0.96). The jackknife procedure revealed that the largest decrease in predictor accuracy resulted from removing UnEx_corr_ scores from the model (Fig. 3C, mean accuracy across 1000 classifications = 88.4%), followed by the total UPDRS I score and LEDD (mean accuracy across 1000 classifications = 88.9%) and the remaining schizotypy scores.

## Discussion

We found that PD patients exhibit lower schizotypy than age-matched healthy controls. This effect is specific to the positive dimension of schizotypy; i.e. UnEx and ImpNon (the latter also tapping specifically into impulsive aspects associated with positive schizotypy). These results are in line with a previous study that observed selective differences in positive schizotypy between PD patients and controls^30^. In this study, however, effects failed to reach significance, probably due to the relatively small sample size (n = 18). These findings indicate that schizotypy, in particular the positive (and impulsive) dimension, is related to neural networks affected by PD. As PD is associated with a depletion of dopaminergic neurons in the substantia nigra and a disruption of basal ganglia-thalamo-cortical processing^25^ and previous studies in healthy controls suggest a role of dopamine and basal ganglia activity in schizotypy^19,26,29,44^, basal ganglia-cortical networks represent one likely candidate for an overlapping neural system. Anatomically, basal ganglia-cortical connections consist of partially overlapping segregated subloops with motor, cognitive and affective components^45^. Despite PD generally being classified as a motor disorder, studies analyzing quality of life suggest that patients feel greatly impaired by non-motor symptoms^46^. We observed that positive schizotypy was associated with affective symptoms, but not cognitive and motor impairment of PD. UnEx and ImpNon scores in PD patients were not related to age, sex, disease severity, disease subtype, motor impairment (UPDRS II and III and H&Y scale) or the dose of dopaminergic medication. UnEx scores correlated positively, however, with specific non-motor symptoms, as measured by the UPDRS I. UnEx scores increased as a function of depressed mood (UPDRS I—item 3) and items related to fatigue (UPDRS I—item 8 and 12), but were not related to cognitive impairment (UPDRS I—item 1). This indicates that positive schizotypy is mediated by affective subloops of the basal ganglia-cortical system. In order to test this hypothesis, future studies could investigate electrophysiological activity in distinct parts of the basal-ganglia-thalamo cortical system during affective tasks as a function of schizotypy (e.g. via depth electrodes in the limbic parts of the subthalamic nucleus in PD patients). Further, one could test the causal role of basal ganglia activity for schizotypy by neuromodulation of the relevant brain areas (e.g. deep brain stimulation of the subthalamic nucleus or thalamus in PD patients). There are at least two explanations for lower positive schizotypy in PD patients. First, positive schizotypy might decrease as a function of neurodegeneration. However, we did not find a relationship between schizotypy scores and disease duration or severity (UPDRS III, H&Y stage). This suggests a second explanation, in which subjects with lower schizotypy exhibit lower baseline dopamine levels and are thus more vulnerable to a disturbance of the dopamine system. If lower positive schizotypy thus predisposes to PD, one would expect to find lower schizotypy prior to disease onset. Future studies could test this hypothesis in patients with rapid eye movement sleep behavior disorder (RBD), a strong predictor of PD that occurs ~ 10–20 years before diagnosis of PD^47^.

Further, we investigated the relationship between schizotypy and PD psychosis. We found that about one third of patients had experienced hallucinations and ~ 11% delusions throughout the course of their disease. This is in line with previous studies reporting similar prevalence in larger cohorts^5,6^. Our analyses revealed that patients, who had previously experienced hallucinations, exhibited higher UnEx scores than patients who had never developed these symptoms. As the UnEx scale includes several questions related to hallucinations, we repeated the analyses after removing these items to avoid circular argumentation (UnEx_corr_). The relationship between UnEx scores and hallucinations remained significant thereafter. Our results indicate that positive schizotypy shares neural networks with regions involved in the generation of hallucinations. Bivariate correlations revealed that none of the other non-motor symptoms (i.e. UPDRS I sub-items) correlated with psychosis (sub-item 2). In particular, there was no relationship between psychosis and cognitive impairment. These results are in line with previous studies that identified severe cognitive impairment as a risk factor for hallucinations in advanced^6,11,15–17^, but not in early PD^10^. We confirm these results in this cohort of patients, who exhibited symptoms corresponding to relatively early stages of PD (median H&Y stage 2).

We further show that schizotypy can contribute to the prediction of PD psychosis. We used two different methods in order to evaluate the predictive value of schizotypy for the development of hallucinations. The calculation of MI revealed that UnEx_corr_ provided the highest independent predictive power for the occurrence of hallucinations. Second, a jackknife procedure based on a supervised machine learning algorithm; i.e. a naïve Bayes classifier, demonstrated that removing UnEx_corr_ from the predictive model resulted in the strongest decrease in prediction accuracy. Together, these results provide converging evidence that the UnEx_corr_ score represents the most powerful predictive value among a range of relevant patient characteristics and disease-related factors. We believe that this predictability is not restricted to our cohort, but generally applicable, as we included patients with a broad range of demographics and clinical traits (Table 1). We did not find any differences in schizotypy comparing patients with and without delusions. This, however, might be partially related to low statistical power due to sample size.

Due to the cross-sectional design of this study, our data neither allows to establish causal relationships between medication and PD psychosis, nor to dissociate between drug-induced and disease-related hallucinations. However, PD psychosis has rarely been reported during the pre-levodopa era^48^ and several studies show that psychosis in untreated PD patients is rare (1–3%)^12–14^. Further, some studies report a relationship between PD psychosis and dopaminergic drug dose and class^16,49–51^. In light of these studies, psychosis measured in this study is likely related to dopaminergic treatment. In line with this interpretation, our machine learning approach showed that removing LEDD from the model resulted in the third largest decrease in accuracy for the prediction of psychosis (after UnEx_corr_ and CogDis). This indicates that the dose of daily dopaminergic medication indeed plays an important role for the generation of psychotic symptoms. However, our mutual information analysis suggests that LEDD is not a strong independent predictor of psychosis. This indicates that LEDD constitutes a crucial factor for the multifactorial pathogenesis of PD psychosis, but exerts its effect as function of other patient characteristics, primarily positive schizotypy. In fact, one previous study in PD patients showed that administration of dopamine agonists is associated with enhanced positive schizotypy^31^. In this study, UnEx scores were, however, not corrected for questions aiming at hallucinations. Thus, this effect could be mediated by measuring acute PD psychosis.

The results of the current study motivate future studies which should apply a longitudinal study design following treatment-naive patients from diagnosis up to several years. In such studies, a similar machine learning classifier could be used based on prospective data in order to validate our findings for clinical use when planning medication schemes for newly diagnosed patients. In addition, one goal of future studies is to develop a compact and specialized inventory based on the most predictive features of the O-LIFE (or another measure of schizotypy) and other highly predictive patient characteristics. This measure might subsequently serve as a concise quantitative risk assessment tool that can be applied in a simple, cost- and time-effective manner by any clinician before starting dopaminergic treatment.

In summary, we show for the first time that PD patients exhibit lower positive schizotypy than healthy controls. This result in combination with positive correlations between affective non-motor symptoms and positive schizotypy in PD suggests an overlap between neural networks mediating these two processes. Further, we show that patients, who had previously developed PD psychosis, exhibited higher positive schizotypy. The calculation of mutual information and a supervised machine learning algorithm provide converging evidence that the predictive power of schizotypy is superior to other patient characteristics and disease-related factors. Together, our results suggest a clinical application of schizotypy assessment, in particular the UnEx scale, for predicting psychosis in PD patients. Considering the burden caused by PD psychosis, our results are highly clinically relevant, as they suggest a means of predicting and thus potentially reducing patients’ dopamine-related side effects when initiating treatment.
